# A technical review of bail-out procedures to place Najuta stent-graft into the ascending aorta

**DOI:** 10.1186/s42155-023-00351-4

**Published:** 2023-02-21

**Authors:** Raffaella Berchiolli, Nicola Troisi, Giulia Bertagna, Andrea Colli, Laura Besola, Roberto Silingardi, Gioele Simonte, Giacomo Isernia, Italian Najuta registry collaborators, Matteo Orrico, Massimo Lenti, Gianbattista Parlani, Gianluigi Fino, Tea Covic, Stefano Gennai, Michelangelo Ferri, Emanuele Ferrero, Simone Quaglino, Antonio Rizza, Gabriele Maritati, Michele Portoghese, Fabio Verzini, Andrea Discalzi, Raffaele Pulli, Aaron Fargion, Stefano Bonvini, Francesco Intrieri, Francesco Speziale, Wassim Mansour, Diego Moniaci, Nicola Troisi, Andrea Colli, Stefano Camparini, Genadiev Genavi, Giovanni Pratesi, Francesco Massi, Stefano Michelagnoli, Emiliano Chisci, Stefano Bonardelli, Massimo Maione, Domenico Angiletta, Sergio Zacà, Gian Franco Veraldi, Luca Mezzetto

**Affiliations:** 1 Vascular Surgery Unit, Department of Translational Research and of New Technologies in Medicine and Surgery, University of Pisa, Cisanello Hospital, Via Roma 67, 56126 Pisa, Italy; 2 Division of Cardiac Surgery, Department of Surgical, Medical and Molecular Pathology and Critical Care, University of Pisa, Pisa, Italy; 3 Department of Vascular Surgery, University of Modena and Reggio Emilia, Nuovo Ospedale S. Agostino Estense, Modena, Italy; 4 Department of Vascular and Endovascular Surgery, University Hospital of Perugia, Perugia, Italy

**Keywords:** Najuta stent-graft, Aortic arch, Thoracic endovascular aortic repair (TEVAR)

## Abstract

**Background:**

The Najuta stent-graft (Kawasumi Laboratories Inc., Tokyo, Japan) is usually easily advanced to the correct deployment position in the ascending aorta thanks to the pre-curved delivery J-sheath with all fenestrations automatically oriented towards the supra-aortic vessels. Aortic arch anatomy and delivery system stiffness could however represent limitations for proper endograft advancement, especially when the aortic arch bends sharply. The aim of this technical note is to report a series of bail-out procedures that could be useful to overcome the difficulties encountered during the Najuta stent-graft advancement up to the ascending aorta.

**Main body:**

The insertion, positioning and deployment of a Najuta stent-graft requires a through-and-through guidewire technique using a .035″ 400 cm hydrophilic nitinol guidewire (Radifocus™ Guidewire M Non-Vascular, Terumo Corporation, Tokyo, Japan) with right brachial and both femoral accesses. When standard maneuver to put the endograft tip into the aortic arch, some bail-out procedures can be applied to obtain proper positioning. Five techniques are described into the text: positioning of a coaxial extra-stiff guidewire; positioning of a long introducer sheath down to the aortic root from the right brachial access; inflation of a balloon inside the ostia of the supra-aortic vessels; inflation of a balloon inside the aortic arch (coaxial to the device); and transapical access technique. This is a troubleshooting guide for allowing physicians to overcome various difficulties with the Najuta endograft as well as for other similar devices.

**Short conclusion:**

Technical issues in advancing the delivery system of Najuta stent-graft could occur. Therefore, the rescue procedures described in this technical note could be useful to guarantee the correct positioning and deployment of the stent-graft.

**Supplementary Information:**

The online version contains supplementary material available at 10.1186/s42155-023-00351-4.

## Background

Among fenestrated endografts, the semi-custom made Najuta stent-graft (Kawasumi Laboratories Inc., Tokyo, Japan) is a useful tool for endovascular specialists’ armamentarium (Sato et al. 2020; Isernia et al. 2023), even in patients with bovine arch (Toya et al. 2018).

The aim of this technical note is to describe a series of bail-out procedures to overcome potential difficulties encountered during the Najuta stent-graft advancement to the ascending aorta.

## Main text

The insertion, positioning and deployment of a Najuta stent-graft requires a through-and-through guidewire technique using a .035″ 400 cm hydrophilic nitinol guidewire (Radifocus™ Guidewire M Non-Vascular, Terumo Corporation, Tokyo, Japan) with right brachial and both femoral accesses. The standard technique consists of advancing the endograft over the through-and-through maintaining tension pulling the wire from cranial and distal accesses, until the endograft tip reaches the arch, then tension is released and a loop is created in the aortic root to facilitate the stent-graft tip advancement up to the desired deployment position into the ascending aorta. If this standard maneuver fails, and the endograft tip cannot be properly advanced into the aortic arch, some bail-out procedures can be applied to obtain proper positioning in the ascending aorta and aortic arch.

### Coaxial extra-stiff guidewire

**Fig. 1:**
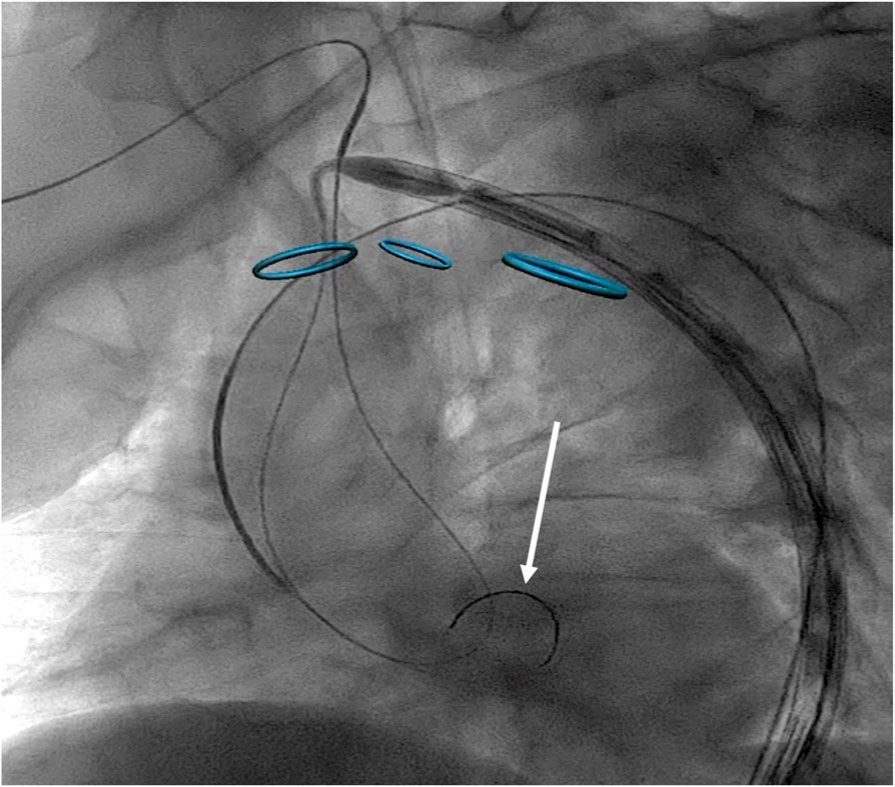
Coaxial extra-stiff guidewire put inside the ascending aorta (arrow)

The first bail-out maneuver consists in positioning a super-stiff guidewire (Lunderquist .035″ extra-stiff 260 cm guidewire; Cook Inc. Bloomington, IN, USA is generally preferred) from the contralateral femoral access (through the introducer in place for the pigtail insertion). The extra-stiff guidewire should be advanced close to the aortic root using a pig-tail catheter to protect the valve. Once the extra-stiff guidewire has been positioned, the stent-graft can be pushed at the same time by using the standard loop technique, facilitating the positioning of the stent-graft tip into the ascending aorta (Fig. 1).

### Long introducer sheath down to the aortic root from the right brachial access

If the coaxial extra-stiff guidewire fails, the second bail-out procedure option is the positioning of a long introducer sheath close to the aortic root from the right brachial access.

This technique consists of exchanging the brachial introducer sheath (usually a 4Fr 11 cm is used to perform the standard through-and-through guidewire technique) with one larger and longer (7-8Fr and at least 70 cm recommended). This maneuver should allow the user to apply a force focused on the aortic root to allow the stent-graft tip advancement into the ascending aorta. Two experienced physicians are needed to perform this procedure: one pushes the introducer sheath onto the aortic root, and the other one pushes the graft forward from the femoral access. Tension on the through-and-through guidewire must be carefully adjusted according to perceived friction and opposition during graft advancement.

A sheath-anchoring rail guidewire can be used to improve the pushability of the long introducer sheath onto the aortic root.

**Fig. 2:**
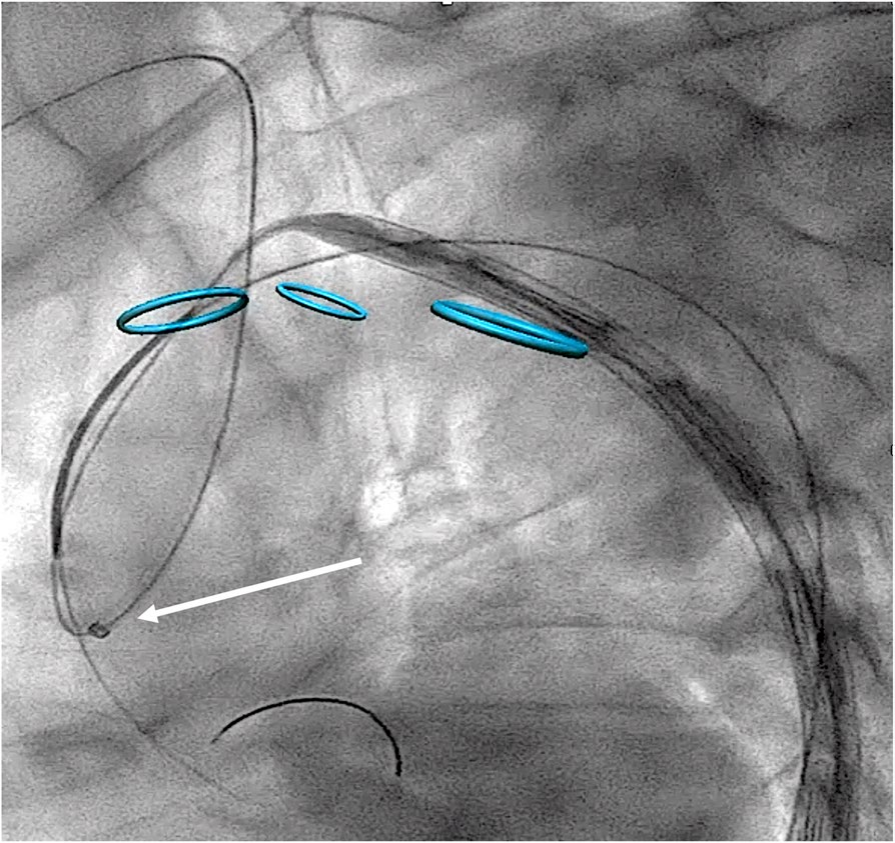
Long introducer sheath from right brachial access down to the aortic root (arrow)

The main disadvantage of this second approach is the need to increase the introducer sheath size of the right brachial access, increasing the risk of periprocedural complications (Fig. 2).

### Balloon inflated inside the supra-aortic vessels

If the second bail-out procedure results are also ineffective, the subsequent maneuver consists of inflating a standard angioplasty balloon inside one of the supra-aortic vessels ostia aiming to provide a smooth surface on the outer arch curvature. Thus facilitates the Najuta stent-graft advancement towards the inner curvature of the aortic arch until the ascending aorta. The diameter of the balloon should be evaluated based on a meticulous preoperative assessment.

**Fig. 3:**
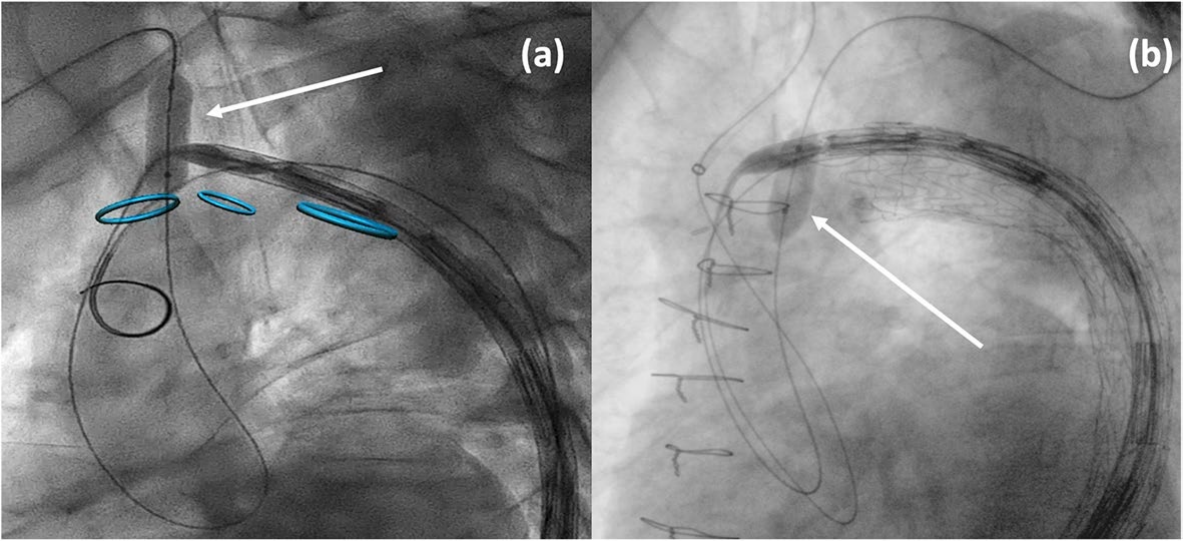
Balloon inflated inside the ostia of the supra-aortic vessels: **a** innominate trunk (arrow); **b** left subclavian artery (arrow)

The first ostium to be used should be the innominate artery since the right brachial access is already available. If the inflation of the ostium of the innominate trunk also fails, it is possible to inflate a balloon on the ostium of the left subclavian artery; to do that it is then necessary to perform a left brachial/axillary access with a percutaneous or surgical approach. The ostium of the left common carotid should be considered as the last option, and it could be reached with a retrograde approach (surgical or percutaneous) (Fig. 3).

Cerebral monitoring is useful to evaluate the cerebral status during the balloon inflations inside the ostia of the supra-aortic vessels.

### Balloon inflated inside the aortic arch

**Fig. 4:**
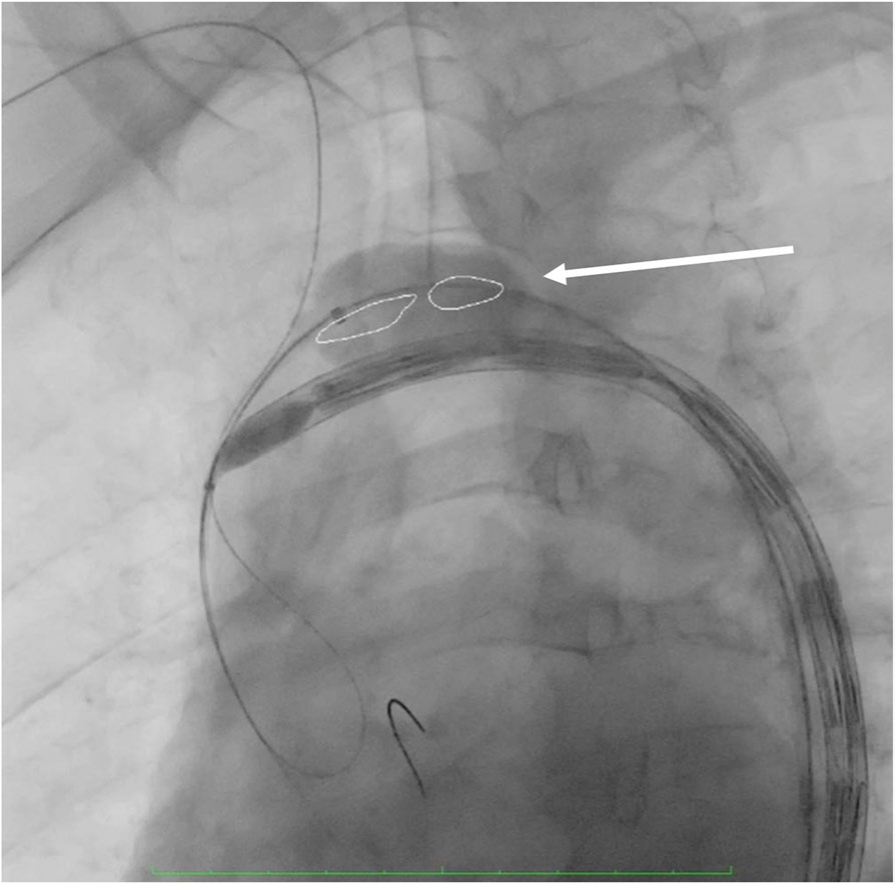
Balloon inflated inside the aortic arch (arrow)

Another technique to be performed is to inflate a large compliant balloon (usually a Coda, Cook Inc. Bloomington, IN, USA) into the aortic arch very close to the Najuta stent-graft tip. The aortic balloon should be inserted from the contralateral femoral access. This bail-out procedure also should be performed by two skilled operators, who simultaneously inflate the aortic balloon turning away the Najuta stent-graft from the aortic arch outer curvature and redirect the tip towards the ascending aorta. However, the proper balloon positioning into the outer curvature is not easy to obtain. This technique is the riskiest since it could lead to cerebral and peripheral embolizations (Fig. 4).

### Transapical access

The last option is a transapical access. A left mini-thoracotomy in the fifth intercostal space is performed exposing the apex of the heart. After transesophageal echocardiography (TEE) control the correct entry site is defined and double pledgeted U-stitch purse strings with 2/0 monofilament suture are placed in the apical-lateral portion of the ventricle as described elsewhere (Colli et al. 2014) (Additional  file 1).

Under fluoroscopy or TEE guidance, the apex is punctured with a standard fluoroscopic needle and a standard 7F introducer sheath is placed and a guidewire is advanced crossing the aortic valve. A goose neck system could be advanced into the ascending aorta and the tip of the guidewire supporting the Najuta stent-graft could be exteriorized from the transapical access. As described in the long introducer sheath technique, a synchronized push and pull from two operators is required to further advance the Najuta stent-graft into the ascending aorta.

## Conclusions

A recent systematic review (Blanco Amil et al. 2021) reported similar numbers of patients treated with fenestrated or branched grafts for aortic arch pathologies; no comparative study has been published in Literature.

Najuta stent-graft offers low complication rates (Sato et al. 2020; Isernia et al. 2023).

Delivery system advancement could present some technical issues, especially in sharp aortic arch anatomy. Fernandez-Alonso et al. (Fernández-Alonso et al. 2020) described a bail-out procedure during a Bolton Relay PLUS by placing a 4F pigtail with a stiff guidewire from the right brachial access in order to perform a through-and-through guidewire technique with a femoral access. Mastrorilli et al. (Mastrorilli et al. 2022) described the sheath-anchoring rail guidewire technique to advance a thoracic stent-graft into the aortic arch.

Another technical issue has been reported during the deployment of a Cook Zenith preloaded F-TEVAR: the through-and-through guidewire entanglement around the delivery sheath, resulting in a device malrotation. A total of four bail-out procedures have been already reported in order to face these situations (Prendes et al. 2021).

However, none of the bail out procedures described in literature has the Najuta as stent-graft. Endovascular repair with Najuta stent-graft is useful as a less-invasive option for high-risk patients who are unfit for open surgery of the aortic arch, even if the perioperative outcomes could be affected by the length of the proximal aortic neck into the ascending aorta.

In this paper, we report several bail-out procedures with the aim to provide a troubleshooting guide for allowing physicians to overcome various difficulties with the Najuta endograft as well as for other similar devices. Once all the endovascular bail-out procedures have been attempted, a transapical access with a through-and-through guidewire technique could be considered.

## Supplementary Information

**Additional file 1.** Transapical access.

## Data Availability

The datasets used and/or analysed during the current study are available from the corresponding author on reasonable request.

## Abbreviations

TEE: Transesophageal echocardiography
F-TEVAR: fenestrated thoracic endovascular aortic repair
