# Supplementary figures and images for: A technical review of bail-out procedures to place Najuta stent-graft into the ascending aorta
Source: CVIR Endovasc. 2023 Feb 21;6:7. doi: 10.1186/s42155-023-00351-4 (PMC9944130; doi:10.1186/s42155-023-00351-4)

## Slide 1
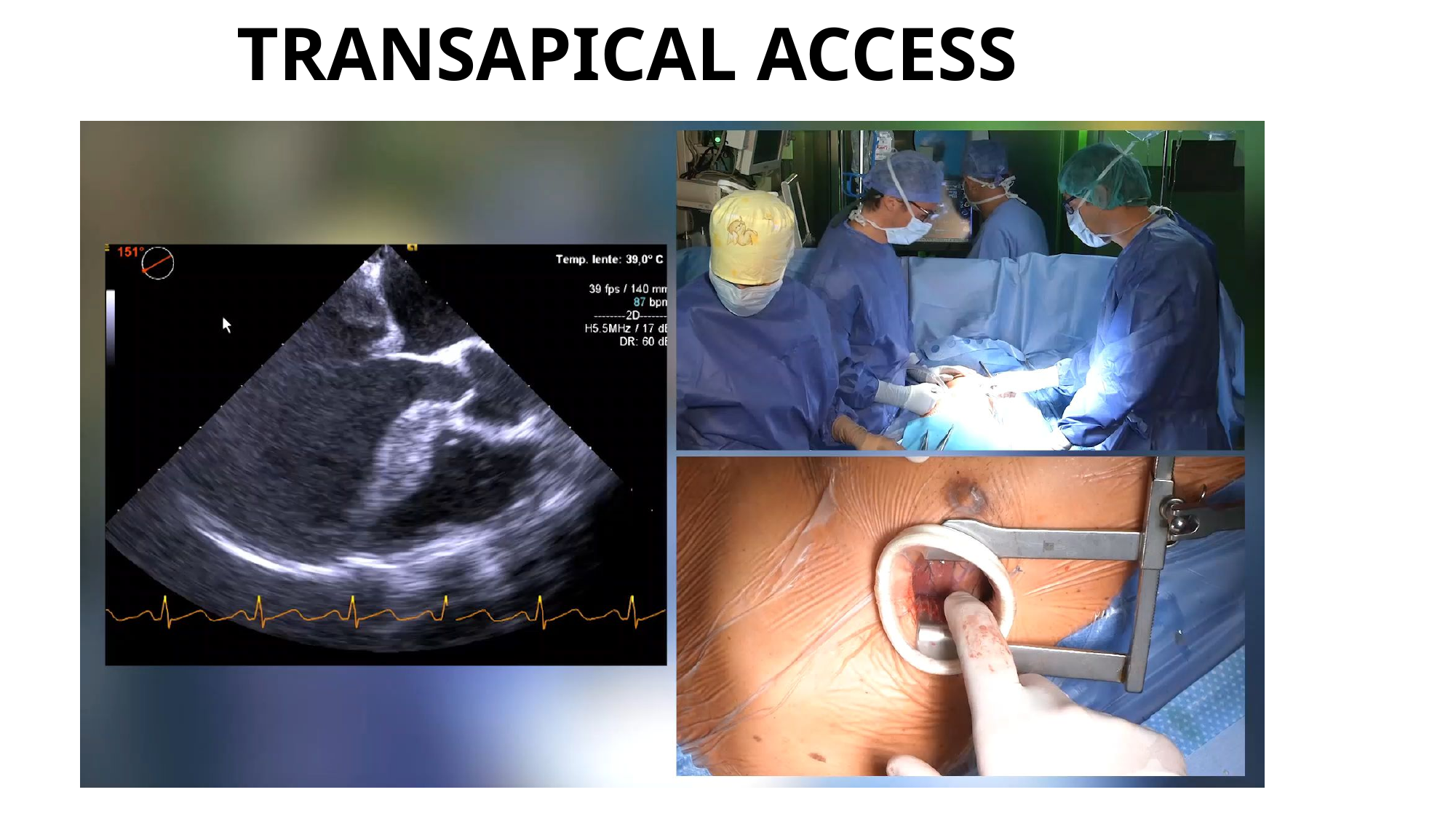

TRANSAPICAL ACCESS

Supplement: Supplementary file 1 — Additional file 1. Transapical access. [file 42155_2023_351_MOESM1_ESM.pptx]
